# Analyzing the Social Construction of Media Claims: Enhancing Media Literacy in Social Problems Classes

**DOI:** 10.1177/0092055X18793493

**Published:** 2019-01

**Authors:** Todd K. Platts

**Affiliations:** 1 Piedmont Virginia Community College, Charlottesville, VA, USA

**Keywords:** media literacy, social problems, content analysis, social constructionism, news analysis

## Abstract

Recent research has called on scholars to develop pedagogical interventions to address issues of media literacy. This teaching note answers that call by describing a media literacy project designed for use in social problems classes. The project acquaints students to the constructionist approach to social problems and the method of content analysis. Guided by the principles of scaffolding, the note discusses how students are guided through a series of readings, assignments, and activities that enables them to analyze how social problems are portrayed in news media. Analysis of student papers and comments reveals an increased understanding of the rhetorical strategies that are commonplace in media coverage of social problems. While designed for social problems, the project can be adjusted and modified for use in other sociology courses.

This teaching note discusses a media literacy project that employs qualitative content analysis and is intended for courses in social problems. While designed specifically for social problems, the same assignment can be adjusted and assigned to other sociology courses—including introductory courses, sociology of the family, culture and media, and race and ethnic relations to name a few. The project serves as a response to a growing number of studies that document issues with media literacy and the impact this has on civic participation and engagement (e.g., Allcott and Gentzkow 2017; Marchi 2012; Martens and Hobbs 2015; Merpert et al. 2018; Stanford History Education Group 2016). The assignment addresses these concerns by having students employ a constructionist analysis to journalistic coverage of a chosen social problem.

The note proceeds by first providing more background on the constructionist approach to social problems. From there, media literacy issues are highlighted. This is followed by a brief look at content analysis and statements regarding the project’s learning objectives. Next, focus shifts to the delivery of the project, including the lectures and readings leading up to it, the activities that ensure student comprehension, a coding workshop, and tips on writing for students. The note concludes with an assessment of student learning.

## The Constructionist Approach to Social Problems

The constructionist approach is a specific framework to the sociological analysis of social problems that examines the process by which troubling conditions become recognized as problems (Spector 1976). It contrasts with the objectivist approach that attempts to definitively demonstrate that certain troubling conditions cause demonstrable harm to society (see Best 2017; Harris 2013). Instead, the constructionist approach pays attention to how certain issues are discussed and understood as social problems through “the process of claims-making” (Best 1987:101). In other words, the focus is on “how claimsmakers bring attention to problems, how the media cover these concerns, the reaction of the public, and how policymakers deal with the issues” (Harris and Best 2013:285). Claims (and claimsmaking), then, constitute a primary concern of constructionist analysis of social problems, as does the history of those claims (Spector 1976). Analysis of claims serves as the locus of concern for the project. Drawing from Stephen Toulmin’s (1958) analysis of arguments, Joel Best (2013) argues that claims can be divided into *grounds, warrants*, and *conclusions*.

Grounds provide the basic facts and nature of a problem (Best 1987, 2013). That is, “they argue that the condition exists and offer supporting evidence” (Best 2017:31). Grounds do not need to be empirically true, they simply must be believed by those receiving them. What counts as a reasonable and relevant grounds varies by society, historical era, and constituency (Best 2013). Grounds in modern-day United States are often established with rhetorical moves that include: a typifying example, a name, and a statistic or statistics. A typifying example is an instance of a problem that illustrates its seriousness (Best 2013, 2017). Because typifying examples attempt to draw attention to problems, they tend to be shocking, melodramatic, and memorable. This means that typifying examples are usually not typical. Typifying examples are then transformed into instances of larger issues with names such as *road rage* (Best and Furedi 2001), *child abuse* (Pfohl 1977), *distracted driving* (Parilla 2013), and *Internet addiction* (Schweingruber and Horstmeier 2013). It is significant to point out that naming is not the same as defining a problem. In fact, claimsmakers tend to prefer broad, vague definitions to capture more cases and make the problem seem larger (Best 2013). Statistics are used to indicate the scope of a problem. While statistics connote accuracy and precision, the numbers used to promote a problem are often exaggerated and/or speculative (Best 2013, 2017).

Where grounds describe a problem, warrants justify taking action to address that problem (Best 1987, 2013, 2017). Accordingly, warrants tend to play on deeply held values and arouse emotional responses. When playing on values, warrants tend to point out a disjunction between a condition and a deeply held cultural value, such as the Civil Rights Movement highlighting the United States’s failure to offer equality of opportunity to its citizens. When playing on emotions, warrants tend to shine light on the plight of vulnerable victims such as women and children (Best 2013). Warrants can also evoke words with powerful connotations such as *epidemic* and *crisis* as a ploy to draw attention to an issue (LeMoyne and Davis 2011).

Conclusions are calls for action to alleviate or erase the social problem (Best 1987, 2013, 2017). Conclusions can include short-range and long-range objectives. With short-range objectives, advocates of a troubling condition can generate awareness of the problem, garner media attention for it, and get people to buy into it. Long-range objectives can involve the passing of social policies and funding programs or research to deal with the problem. While grounds, warrants, and conclusions are the elements comprising social problems claims, these elements are not always readily apparent in claims. On occasion, inference is necessary.

The project attempts to help students recognize the construction of social problems as they are articulated in news media. While this article describes a news analysis project similar to those already in use in sociology classrooms, its focus on deconstructing claims and claimsmaking adds a new wrinkle to an old pedagogical practice. The exercise gives students a more thorough understanding of how news stories on social problems are rhetorically structured (Lowry 2016) and thereby contributes to the growing pedagogy of media literacy. It also offers a form of learning by doing in one of social problems’ major frameworks (American Sociological Association 2016).

## Media Literacy

Though there is near unanimity on the importance of media literacy and the necessity of efforts to improve it, scholars have yet to come to a widely agreed on definition of the term (Maksl, Ashley, and Craft 2015; Maksl et al. 2017; Potter 2013). Also, debate exists on what media literacy education should emphasize. Should educators teach students about the industries producing media and their messaging and effects on the populace? Should they focus on existing media preferences to deepen critical analytic skills? Or, should instructors promote civic outreach via media production activities (for a review of these debates, see Martens 2010). To cut through this impasse, I follow Malik et al. (cited in Maksl et al. 2017:229) by defining media literacy in terms of the ends it is meant to achieve. In this case “empowered citizens,” which entails: “1) an understanding of the role news plays in society . . . 2) motivation to seek out news . . . 3) the ability to find/identify/recognize news . . . 4) the ability to critically evaluate news . . . 5) the ability to create news.” The project most demonstrably develops student ability to critically evaluate news and arguably facilitates a broader understanding of the role news plays in society and sharpens student news gathering competences.

Studies have routinely documented poor media literacy among students and the general population, a fact that has only been exacerbated with the advent of social media (Allcott and Gentzkow 2017; Maksl et al. 2015; Marchi 2012; Stanford History Education Group 2016). To quote the Stanford History Education Group (2016:4), “Overall, young people’s ability to reason about the information on the internet can be summed up in one word: bleak.” People have been shown to be easily duped, especially with content that aligns with their own biases (Allcott and Gentzkow 2017). Fortunately, pedagogical media literacy interventions have been demonstrated to show “positive effects on outcomes such as media knowledge, criticism, perceived realism, influence, behavioral beliefs, attitudes, self-efficacy, and behavior” (Maksl et al. 2017:230).

## Content Analysis

The primary method of pedagogical intervention and discovery for this project is qualitative content analysis. Content analysis entails the study of social artifacts such as comics, film, and art through the development of a formal coding scheme where the intent is the “drawing of empirical and theoretical inferences from the chosen text type” (Churchill 2014:255). The method can be approached inductively, that is, lacking formal coding scheme prior to analysis. It can also proceed deductively, that is, with a formal coding scheme prior to analysis. Social artifacts contain both manifest and latent content. Manifest content are those elements that can be objectively tabulated in an artifact, such as the word count of an article, number of people quoted in it, and appearance of certain words (Neuendorf 2002). Latent content, by contrast, are the more interpretive and implicit elements in an artifact, such as political slant, author motives, and how the artifact reflects its cultural milieu (Neuendorf 2002). As much of the project entails analysis of latent content, the project follows Taylor’s (2003:303) recommendation of “corroborative techniques, such as using independent coders or providing detailed excerpts from the data, which support the stated interpretations.”

Regardless of whether the researcher utilizes an inductive or deductive approach or codes latent or manifest content, all content analysis requires a similar analytic process, including “formulating the research questions to be answered, selecting the sample to be analyzed, defining the categories to be applied, outlining the coding process and coder training, implementing the coding the process, determining the trustworthiness, and analyzing the results of coding process” (Hsieh and Shannon 2005:1285). Obviously, teaching all this to research novices is a tall task. Indeed, instruction of the method has been uneven, with it being taught across a semester in sociology of media courses (Papademas 1983), within a string of lectures in criminology classes (Finley 2004), as part of a single lecture on gender stereotypes (Taylor 2003), and as part of a larger lecture on research methods (Messinger 2012). The approach taken in the following is a middle-ground one, taking several lectures of a class to teach the social constructionist framework for social problems and the method of content analysis. The middle-ground approach affords the achievement of specific learning objectives and a more in-depth engagement with them while heeding Papademas’s (1983:394) warning of “taking away from other teaching modes.”

## Learning Objectives/Outcomes

To achieve the learning outcomes of the exercise, the following material should be addressed beforehand: the constructionist approach to social problems, content analysis, and influence of media on social problem claims. Though other learning objectives may be listed, the following are considered the most pertinent: Demonstrate (1) an understanding of the constructionist approach to social problems through an application of it, (2) a working knowledge of qualitative content analytic methods through a constructionist analysis of news articles, and (3) an understanding of news media through identifying common rhetorical strategies in establishing social problems. Successful completion of the project will not only acquaint students with a dominant paradigm of social problems research but will ideally also make them more critical consumers of media. Further, the learning objectives call on students to analytically deconstruct claims that empower students by augmenting their ability to evaluate news claims.

## The Project and Background

The project occurs across the first third of the semester. To date, the author has taught the course every spring as a class that meets twice a week for 75 minutes per session for 15 weeks. It is also significant to note that the author teaches at a small- to midsize community college in the South. This means that student enrollment is relatively low (the course is capped at 30) and that many students come to the class with little background in sociology. Though not common, some students in the class are co-enrolled in remedial writing and reading classes. This is brought up to demonstrate that students do not need an advanced level of sociological knowledge to adequately complete the project.

The project has several parameters. Students are asked to select a “social problem” of their choosing and analyze how this problem is constructed as such in news media. Because of time constraints, pointers related to the constraints of the project, such as making sure to select a sample with a limited timeframe and collect stories from a limited number of sources, are given to students.^1^ Once a problem has been identified and approved by the instructor, students are instructed to select a sample of 10 articles. Even though this sample may prevent generalizability of findings, the restriction keeps the workload of the project manageable (cf. Rushing and Winfield 1999). Since the project teaches critical media literacy, the ideological biases of the news sources that students use does not present a significant issue (Bonomo 1987; Spector 1976).

## Delivery

Several activities help to ensure student comprehension of the constructionist approach to social problems as well as the competency needed to complete the assignment. The nature and ordering of the activities are informed by the idea of scaffolding. Briefly, scaffolding is “the temporary assistance that teachers provide for their students to assist them to complete a task or develop new understandings, so that they will later be able to complete similar tasks alone” (Hammond and Gibbons 2005:9). Scaffolding operates on the notion that carefully sequenced tasks and activities incrementally build and facilitate student learning and academic autonomy (Peyrefitte and Lazar 2018). The tasks (or steps) highlighted in the following slowly build student knowledge of the skillsets most needed for the project.

### Step 1: Lectures and Supporting Material and Activities

On the first day of class, students are introduced to the basic requirements for the course, including the project discussed in this teaching note (there are three projects altogether). The constructionist paradigm is introduced during the third class meeting. As Joel Best’s (2017) *Social Problems* is the assigned text, students are asked to read the second chapter before coming to class. Instructors using a different textbook or set of texts can use Best’s (2013) “Constructionist Social Problems Theory,” which succinctly describes the framework. The lecture is straightforward, making sure to go over the concepts and deriving relevant examples to illustrate them.

Before the next class, students are assigned a reading on content analysis. While there are many worthwhile readings on the method, Brendan Churchill’s (2014) summary of it is approachable, uses understandable examples, and is brief. Since the project codes for grounds, warrants, and conclusions, students are informed that they will be doing a deductive analysis.

The next class contains two activities, both designed to get practice at content analysis. For the first, two newspaper articles are distributed. One addresses teen drinking and driving as a problem, the other road rage (almost any article will be acceptable, so long as it purports to address a social problem). Then, as a class, a line-by-line coding on the presence of grounds, warrants, and conclusions for each article is performed. For the exercise, instructors should try to allow students to do as much talking as possible, probing them to answer why this line is a ground, warrant, or conclusion. Inevitably, disagreements emerge, which allows deeper discussions of the nature of content analysis. As Stalp and Grant (2001:201) note, “since students typically disagree on some classifications, they gain insights into how personal history and biography are linked to perceptions of reality.” Moreover, students are instructed on the importance of intercoder reliability and the idea that coding categories will rarely reach 100 percent agreement between different coders. The second activity is an in-class small group writing assignment wherein students code a story written by the instructor. (The author has students code a story on a fictitious zombie outbreak.) Students code each line of the story, and where disagreements occur, students are encouraged to document them.

Pursuant to the aforementioned lessons, students are instructed to start thinking about a social problem to analyze. Students are asked to vet project ideas with the instructor to ensure appropriateness (i.e., has their topic been constructed as a social problem) and narrow their focus. Once students have selected an appropriate problem for analysis, they are asked to collect their sample. Students are given a few weeks to collect their sample as other lessons in the course are pursued.

### Step 2: Supplemental Lectures and Data Collection

Just before the workshop for the project, students are given a lecture on the impact media has on social problem claims (Best 2013, 2017). Here, students are reminded that the nature of news work necessarily alters the nature of claims. Sometimes this altering has nothing to do with the political slant of the media agency or individual reporter. The reporting of news is constrained by economic concerns (the cost of producing a story, hiring creative personnel), space constraints (broadcast minutes, word counts), and deadlines. What is more, the advent of cable news and, now, social media has allowed for narrowcasting (or audience segmentation) wherein coverage of social problem claims is catered to a particular audience (e.g., Iyengar and Hahn 2009; Oyedeji 2010).

### Step 3: Workshop

The workshop for the project lasts an entire class period. Students are instructed to bring at least two copies of their sample. In class, they are placed in groups of two or three. The workshop starts with each student coding five articles in their sample, marking their codes on their articles. After coding the five articles, students will exchange blank duplicate copies of the same articles for their partner to code. Where disagreements exist, students are expected to dialog to see if resolving them is possible. In instances where resolution is not achieved, students are asked to make note of the disagreements and document them in their papers. Time permitting, students can code the entirety of their sample. During the workshop, instructors should act as facilitators. Some students will need to be prodded more than others. The idea is to get students to do their own coding, not for the instructor to do it for them. It is advisable to have students revisit their notes on grounds, warrants, and conclusions and explain their thought processes. It is also important to get partners to converse with each other to gain a further appreciation of the research process (Stalp and Grant 2001). Students should also be encouraged to take notes during the workshop, including the nature of the grounds, warrants, and conclusions they identified, to help in the writing of the paper.

### Step 4: Writing

When students have completed the workshop, they are given instructions on how to write the accompanying paper. Generically, students are advised to organize their analysis around the major components of social problem claims, that is, separate sections on grounds, warrants, and conclusions. As one of the purposes of the project is to get students acquainted with data analysis (and not full-blown academic research per se), the task of the written project can be achieved in four to seven pages. With data collection and coding complete, students are given two weeks to write up their results.

## Assessment of Student Learning Outcomes

For knowledge of best pedagogical practices to advance and contribute to “pedagogical culture,” new interventions must be subjected to empirical scrutiny (Peyrefitte and Lazar 2018). In the project detailed previously, the most pertinent learning objectives were operationalized as applying and therefore understanding the constructionist approach to social problems, performing a qualitative content analysis utilizing the constructionist perspective, and deconstructing common rhetorical strategies used to construct social problems in news media. To demonstrate these learning objectives, students were expected to address of number of points in their projects. When showing knowledge of the constructionist approach to social problems, students should acknowledge that they are assessing claims or are aware that debate over the nature of their selected problem has changed across time, among other strategies. In displaying aptitude in qualitative content analysis and constructionist analysis, students are expected to discuss their employment of the method in their projects as well as a discussion of grounds, warrants, and conclusions as they relate to their chosen issue. Finally, proficiency in deconstructing common rhetorical strategies in the media construction of problems is established when students identify and analyze themes or patterns in the reporting of their issue as they relate grounds, warrants, and conclusions.

To assess student acquisition of learning objectives, completed projects were qualitatively analyzed from 25 students enrolled in social problems during the spring 2018 semester. A total of 29 students were enrolled in the class, with 2 failing to finish the project and 2 opting to participate in service learning instead. Students were slightly demographically dissimilar from the college’s population. The majority of students in the class were male (52 percent) compared to 43 percent of the school’s total. The median age (21) and portion of nonwhite students (30 percent), however, were more compatible with college averages (Piedmont Virginia Community College N.d.). The instructor has utilized the project since spring 2016, but other semesters were ruled out of the analysis due to incomplete data sets. As well, the steps outlined in the delivery section were only followed in the analyzed semester. Small adjustments were added that improved completed projects.

Overall, the vast majority of students (21/25) correctly completed the project and demonstrated its learning objectives. The manner in which students met the learning objects ranged from developing skills (e.g., the paper was structured around grounds, warrants, and conclusions; patterns were identified; and some analysis of those patterns was offered) to mastery (e.g., paper was correctly structured, patterns were identified, those patterns were thoroughly analyzed, and concepts such as typifying examples were identified). Students who did not correctly complete the project often struggled to distinguish the constructionist approach from the objectivist approach to social problems. Instead of analyzing the claims offered in their stories, this small set of students summarized their stories as if they were attempting to convince the reader their chosen issue was a problem. In general, however, students were able to demonstrate understanding of key components of the constructionist paradigm. Specifically, students were able to sociologically analyze news texts, not just summarize them. Their papers show a methodological literacy (learning about new methods and data) and methodological competence (development of analytic skills) (Peyrefitte and Lazar 2018). In so doing, students showed nascent development of methodological skills required for more advanced research. Student response and engagement suggests the project is also capable of stimulating intellectual curiosity in and critical analysis of current events. That is, students can become more critical and proactive consumers of media, as suggested in their comments after the assignment: “Overall, I see Project 1 as a useful tool that allowed me to better understand how context relates to social problems claims and the role that media plays in making those claims.” Likewise, while taking a strict constructionist stance risks “skirting the need to evaluate the content of claims about social problems” (Lowry 2016:179), many students checked the veracity of social problem claims and even reported their fact checking efforts. One shortcoming of the assessment of student learning is the lack of pretest measure of student knowledge prior to completing the assignment.

As the author has refined the project, steps have been added, such as the in-class article analysis and writing tips. In post-project comments, students have responded positively to the steps: “The class exercises gave me more confidence in preparing to write my analysis on the articles” and “The workshop was especially productive because it required us to do the initial research for the project, and it allowed us to employ class lessons.” The quality of completed projects demonstrates the effectiveness of the author’s attempt to scaffold the lessons and activities leading to it.

## Conclusion and Discussion

The project takes a fair amount of planning on the part of the instructor but can be easily implemented into a course. The major benefit is that students integrate class lessons with practical research analysis. As well, students learn how to evaluate media information with sociological skepticism. To quote Deborah Lowry (2016:181), “simply recognizing the regularity with which [students] are exposed to formulaic claims about social problems is an important and typically eye-opening experience for many students,” which was echoed by students: “Before I understood what the claimsmaking process was, I would have just read news articles without understanding how I was being guided by the author with the use of different claimsmaking techniques.” Moreover, the project described in this article has been useful in guiding students through one of the most difficult aspects of learning to think sociologically: to be an active producer of knowledge.

## Notes

1 It is not uncommon for constructionist studies of social problems to span decades for analysis (e.g., Jerolmack 2008; Parilla 2013).
